# Association between serum level of vitamin D and lipid profiles in type 2 diabetic patients in Iran

**DOI:** 10.1186/2251-6581-13-7

**Published:** 2014-01-07

**Authors:** Ahmad Saedisomeolia, Ehsaneh Taheri, Mahmoud Djalali, Ali Malekshahi Moghadam, Mostafa Qorbani

**Affiliations:** 1 Department of Cellular and Molecular of Nutrition, School of Nutritional Sciences and Dietetics, Tehran University of Medical Sciences, Tehran, Iran; 2 Endocrinology and Metabolism Research Center, Endocrinology and Metabolism Clinical Sciences Institute, Tehran University of Medical Sciences, Tehran, Iran; 3 Faculty of Veterinary Medicine, University of Tehran, Tehran, Iran; 4 Department of Public Health, School of Public Health, Alborz University of Medical Sciences, Karaj, Iran; 5 Department of Epidemiology, School of Public Health, Iran University of Medical Sciences, Tehran, Iran

**Keywords:** Diabetes mellitus, Lipid profile, Vitamin D

## Abstract

**Background:**

It is suggested that vitamin D deficiency is associated with cardiovascular disease (CVD) via its effect on lipid profiles. The objective of this study was to determine the association between fasting serum levels of 25(OH) D and lipid profiles in patients with type 2 diabetes.

**Methods:**

This cross-sectional study was conducted on 108 type 2 diabetics. Patients were selected randomly among members of the Iranian Diabetes Association according to study criteria. Fasting concentration of 25(OH) D, calcium, phosphorus, parathyroid hormone (PTH) and lipid profiles (including triglyceride (TG), high-density lipoprotein (HDL), low-density lipoprotein (LDL), and total cholesterol) were measured.

**Results:**

The mean serum levels of 25-hydroxyvitamin D (25(OH) D) and PTH were 53.41 ± 33.25 nmol/l and 40.24 ± 18.24 pmol/l, respectively, in type 2 diabetic patients. Prevalence of vitamin D deficiency was 58.34% and vitamin D sufficiency and insufficiency combined was 41.66%. Although in diabetic patients with vitamin D deficiency, serum levels of total cholesterol, TG, and LDL were higher and HDL was lower compared to patients with vitamin D sufficiency, this association was statistically significant only for serum level of TG (145.91 ± 79.00 *vs*. 122.95 ± 55.82 mg/dl).

**Conclusions:**

The results of present study show that serum concentrations of 25(OH) D were inversely associated with TG. More interventional studies are needed to confirm the relationship between serum concentration of vitamin D and lipid profile in patients with type 2 diabetes.

## Introduction

The incidence of type 2 diabetes mellitus (T2DM), a common chronic and non-communicable disease, is increasing at an alarming rate worldwide [1]. The World Health Organization (WHO) predicts that the global prevalence of type 2 diabetes will increase from 171 million people in 2000 to 366 million in 2030 [2]. T2DM is associated with a high risk of cardiovascular disease, blindness, nephropathy, and neuropathic complications [3,4]. The Iran Multi-Center Osteoporosis Study has estimated the prevalence of mild, moderate, and severe vitamin D deficiency as 47.2%, 45.7%, and 44.2% in the age groups <50, 50–60, and ≤60 years, respectively among women, and 54.2%, 41.2%, and 37.5%, respectively among men in the same age groups. Vitamin D is a fat soluble vitamin with well-known functions in bone homeostasis and metabolism. Research carried out over recent decades indicates that vitamin D deficiency plays an important role in many non-skeletal diseases [5] such as hypertension [6], CVD [7,8], type 1 and 2 diabetes [8], immune disorders, osteoporosis, and cancers [9]. The vitamin D receptor (VDR) is found within various tissues and the α1-hydroxy enzyme (which converts 25-hydroxyvitamin D (25(OH) D) to 1, 25-(OH) 2-D, the active form of vitamin D) is found locally [10].

Many cross-sectional and interventional studies have demonstrated that vitamin D deficiency is associated with impaired glucose tolerance and diabetes mellitus [11-13]. Therefore, serum concentrations of 25(OH) D are lower in T2DM patients compared with healthy controls. There are several mechanisms proposed to explain the inverse relationship between vitamin D and type 2 DM. Vitamin D, via both genomic and non-genomic pathways, has direct and indirect effects on insulin secretion, β-cell function, and insulin resistance [14,15]. On the other hand, cardiovascular disease is the most common cause of mortality in type 2 DM. Researchers have confirmed that vitamin D plays an important role in endothelial function, blood pressure control, calcification of the coronary vasculature, increased vascular resistance, and prevention of CVD [16]. The effect of vitamin D on regulation of the lipid profile is one of the proposed mechanisms for the relationship between vitamin D deficiency and CVD. The aim of our study is to determine the association between serum level of vitamin D and lipid profiles, including serum concentrations of cholesterol, TG, HDL, and LDL, in type 2 diabetic patients.

## Methods

In this cross-sectional study, 108 type 2 diabetic patients with age of 20–80 years from the Iranian Diabetes Association and National Iranian Oil Company (NIOC) - Central Hospital were selected via random sampling. Exclusion criteria included pregnancy, lactation, use of drugs affecting the lipid profile or calcium and bone metabolism, chronic disorders of the liver or kidney, endocrinology disorders such as hypo- or hyperthyroidism and hyperparathyroidism, smoking, insulin injection, use of anticonvulsive drugs, and vitamin D or calcium supplementation. To decrease the seasonal variability in biochemical determinations, our sampling was performed between April and June. A written consent form (approved by the Ethics Committee of Tehran University of Medical Sciences) was signed by all participants. After overnight fasting, 10 ml of peripheral blood was withdrawn. Blood samples were centrifuged at 3000 rpm for 10 min and stored at -20°C. Serum levels of 25(OH) D were measured using the Chemiluminescence Immune Assay method or CLIA (Diasorin, Stillwater, MN) [17], which measures both 25 hydroxyD2 and D3 vitamin D. The normal range of 25(OH) was 6–54 ng/ml (15-135 nmol/l) using this method. Serum levels of PTH were measured using an RIA kit (CIS Biointernational, France) with a normal reference range of 8–79 pg/ml. Serum calcium and phosphorus levels were analyzed using a Pars Azmoon kit (Pars Azmoon Co., Tehran, Iran). Normal ranges of calcium and phosphorus were defined as 8.6–10.3 mg/dl and 2.5–5.0 mg/dl, respectively. Fasting serum glucose was measured using glucose-oxidase with a Pars Azmoon kit (Pars Azmoon Co., Tehran, Iran) and serum insulin was measured using a radioimmunoassay method (Biosorce kit, Denmark). HbA1C was determined using Nyco CardReader ІІ analyzer according the procedure provided [18]. Plasma total cholesterol, HDL-C, and triglyceride concentrations were measured in duplicate using enzymatic kits, standardized reagents, and standards (Pars Azmoon Co., Tehran, Iran). LDL-C concentration was calculated using the Friedewald equation [19]. Anthropometric data including weight and height were measured using a Seca scale (Seca 725; GmbH & Co., Hamburg, Germany) with subjects wearing light clothes and no shoes. Weight and height were measured to the nearest 100 g and 0.5 cm, respectively. Body mass index (BMI) was defined as weight (kg) divided by height squared (m^2^). A 25(OH) D level of less than 50 nmol/L was considered as vitamin D deficiency and levels of equal and more than 50 nmol/L as sufficient [20]. All continuous values are expressed as mean ± SD and categorical variables are presented as percentage. The Student’s *t*-test was employed to compare differences between the means of continuous variables. Multiple linear regression analysis was applied to assess the association of 25(OH) D (as independent variable) with each component of the lipid profiles (as dependent variables) in three models: Model I, crude; Model II, adjusted for age and gender; and Model III, additionally adjusted for BMI. The results of linear regression are presented as B (SE). P-values less than 0.05 were considered statistically significant. Data were analyzed by SPSS statistical software (version 16.0; SPSS Inc., Chicago, IL).

## Results

In our study, 108 individuals with type 2 diabetes were (50.9% men and 49.1% women) participated. The mean serum levels of 25-hydroxyvitamin D (25(OH) D) and PTH were 53.41 ± 33.25 nmol/l and 40.24 ± 18.24 pmol/l, respectively in type 2 diabetic patients. The prevalence of vitamin D deficiency was 58.34% and vitamin D sufficiency and insufficiency combined was 41.66% in patients with type 2 diabetes. Table 1 presents the characteristics of participants according to vitamin D status. In general, the mean levels of TG, FBS were significantly higher and calcium was lower in diabetic patients with vitamin D deficiency compared to patients with sufficient and insufficient levels of vitamin D. Our data revealed that serum levels of 25(OH) D had an inverse, but not significant, association with TG and total cholesterol and a positive correlation with HDL-C and LDL-C after adjusting for confounding variables (Table 2).

**Table 1:** Characteristic of participants by vitamin D status

**Variable**	**Sufficient of vitamin D vitamin D ≥ 50 nmol/L n = 45**	**Deficiency of vitamin D vitamin D < 50 nmol/L n = 63**	**Overall n = 108**	**p-value**
Age	47.63 ± 11.80	47.66 ± 12.38	47.65 ± 12.08	0.74
Gender (M/F) (%)	62.2/37.8	42.9/57.1	50.9/49.1	0.25
Weight (Kg)	75.95 ± 13.38	76.73 ± 13.24	76.42 ± 13.23	0.34
BMI (Kg/m^2^)	26.33 ± 6.99	28.73 ± 5.10	27.76 ± 6.01	0.43
FBS (mg/dl)	129.47 ± 53.38	145.92 ± 75.66	138.80 ± 67.14	0.01
HbA1c (%)	5.91 ± 1.56	6.24 ± 2.00	6.10 ± 1.82	0.10
Insulin	11.48 ± 7.98	12.51 ± 8.20	12.08 ± 8.09	0.97
Calcium (mg/dl)	9.02 ± 0.69	8.85 ± 0.55	8.96 ± 0.50	0.07
Phosphorus (mg/dl)	3.58 ± 0.36	3.66 ± 0.30	3.62 ± 0.33	0.39
PTH (pmol/l)	40.36 ± 16.48	40.16 ± 19.50	40.24 ± 18.24	0.16
Cholesterol (mg/dl)	182.00 ± 37.58	189.89 ± 43.35	186.5 ± 40.96	0.44
TG (mg/dl)	122.95 ± 55.82	145.91 ± 79.00	136.14 ± 70.68	0.01
HDL (mg/dl)	34.83 ± 8.67	34.14 ± 8.42	34.43 ± 8.49	0.85
LDL (mg/dl)	109.36 ± 32.29	113.61 ± 30.08	111.84 ± 30.93	0.71

P < 0.05 was significance.

**Table 2:** **Regression analysis of 25**(**OH**) **D as independent variables and parameters of lipid profile in diabetic patients**

	**Model I**		**Model II**		**Model III**	
	**B (CI 0.95)**	**P-value**^**‡**^	**B (CI 0.95)**	**P-value**^**‡**^	**B (CI 0.95)**	**P-value**^**‡**^
TG	0.02 (-0.05 – 0.10)	0.67	-0.02 (-0.05 – 0.11)	0.86	-0.03 (-0.04 – 0.11)	0.21
TC	0.08 (-0.31 – 0.14)	0.73	0.10 (-0.34 – 0.13)	0.83	0.12 (-0.32 – 0.10)	0.58
LDL-C	0.09 (-0.18 – 0.37)	0.74	0.11 (-0.18 – 0.41)	0.87	0.12 (-0.15 – 0.39)	0.70
HDL-C	0.19 (-0.34 – 0.73)	0.60	0.22 (-0.36 – 0.80)	0.94	0.25 (-0.31 – 0.82)	0.81

B: Unstandardized Coefficients, CI: Confidence Interval.

Model 1: crude.

Model 2: adjusted for age, gender.

Model 3: adjusted for age, gender, BMI.

^‡^: Regression test P-value.

25(OH) D: 25-hydroxy vitamin D (ng/mLTC: total cholesterol (mg/dL); HDL-C: high density lipoprotein cholesterol; LDL-C (mg/dL): low density lipoprotein cholesterol (mg/dL); TG: triglycerides(mg/dL).

## Discussion

The prevalence of vitamin D deficiency was 58.34% and vitamin D sufficiency and insufficiency was 41.66% in patients with type 2 diabetes. Our study findings indicate that there is a negative, but non-significant, relationship between serum levels of 25(OH) D and that of TG in diabetic patients. Few studies have been carried out on the relationship between serum levels of vitamin D and lipid profiles. Ford and colleagues, in their NHANES ІІІ study, found a negative association between serum levels of 25(OH) D and TG in patients with hypertriglyceridemia. However, this relationship was not observed with regard to HDL cholesterol in healthy subjects [21]. Rejnmark and colleagues performed a study among 82 healthy postmenopausal women who had been treated with either 40 mg/day Simvastatin or a placebo for 1 year, in which vitamin D, TG, and LDL levels were measured at baseline and after 26 weeks of treatment [22]. In this study, Simvastatin showed no effect on vitamin D status, but decreased the serum levels of TG and LDL. These results suggest that serum concentration of TG is inversely associated with serum level of 25(OH) D [22]. In contrast, Chiu showed no relationship between serum levels of 25(OH) D and TG or HDL cholesterol in healthy subjects [15].

There is only one review article that addresses the relationship between serum levels of 25(OH) D and lipids. In this analysis, 22 cross-sectional studies and 10 placebo-controlled interventional studies were identified in a 2009 search of PubMed [23]. In all cross-sectional studies, serum levels of 25(OH) D were found to have a direct relationship with HDL cholesterol. In addition, all studies reported an inverse association between serum levels of 25(OH) D and TG. There is no general agreement on the effects of 25(OH) D on serum levels of TG in interventional studies with supplementation of vitamin D. Although a positive association was observed in some studies, other studies showed an inverse relationship between serum levels of 25(OH) D and TG [23]. In all interventional studies on the relationship between vitamin D and lipids, hyperlipidemia had been considered as inclusion criteria and none of these studies had sufficient power to evaluate this relationship. In only one study was a significant effect seen, with an 8% (0.28 mmol/L) increase in serum LDL-C and a 16% (0.22 mmol/L) decrease in serum TG in those given vitamin D as compared to the placebo group [24]. It was suggested that vitamin D has both direct and indirect effects on modifying the lipid profile and that the effect of vitamin D on decreasing serum levels of TG may occur through regulatory action that increases the activity of lipoprotein lipase in adiposity [25]. Several mechanisms are suggested to explain the effect of calcium on lipids, including its reducing role in fatty acid absorption via the formation of insoluble calcium–fatty complexes in the gut. By decreased absorption of fat, particularly saturated fatty acids, it is expected that serum levels of total and LDL cholesterol will be reduced [26]. In addition, calcium can increase the conversion of cholesterol to bile acids due to its ability to bind with bile acids [27]. However, the effect of enteric calcium on lipid absorption is very limited, and it does not have a significant effect on lipid profiles [28].

On the other hand, *in vitro* studies have demonstrated that PTH decreases lipolysis (through increasing the level of cytosolic calcium) [29] and increases the expression of fatty acid synthesis [30].

Garry John and colleagues conducted a study on 170 UK Bangladeshi adults (69 men and 101 women) with no history of diabetes or other chronic disease. Their data showed that the serum level of 25(OH) D is an independent predictor of fasting apolipoprotein A_1_. However, no relationship was observed between 25(OH) D and TG or HDL cholesterol [31]. Apolipoprotein A_1_ is an essential part of HDL cholesterol that acts as a scavenger of cholesterol from tissues and transports it to the liver.

Exposure to sunlight and adequate consumption of foodstuffs naturally rich in vitamin D (liver, fatty fish, dairy products, and egg yolk) are the main requirements for normal vitamin D levels in humans [32]. Dermal synthesis of vitamin D through exposure to ultraviolet compounds from natural sunlight can provide 80–100% of human vitamin D requirements [33].

Dietary vitamin D is incorporated with other lipids in chylomicrons and is absorbed via the lymphatic system. The results of our study showed a statistically non-significant positive association between serum levels of 25(OH) D and total and LDL cholesterol. There are several explanations for these observations. First, the prevalence of vitamin D deficiency is very common in Iran, similar to other countries in the Middle East, because of inadequate consumption of foodstuffs rich in vitamin D and limited exposure to sunlight (for cultural reasons) [34]. Second, the main source of vitamin D in our country is the consumption of animal-based foodstuffs that contain other lipids in addition to vitamin D.

In our study, the maximum duration of illness was 5 years in type 2 diabetic patients. Therefore, the relationship between serum levels of 25(OH) D and lipids may be affected by increased disease duration in these patients.

According to other studies, it is suggested that vitamin D deficiency is associated with changes in serum levels of apolipoproteins, but not with fasting levels of lipids. Therefore, more studies are needed to confirm the possible effects of vitamin D on serum concentrations of lipids.

This study has some limitations, the main one being its cross-sectional nature, with no causality effect to report. Variation in the polymorphisms of vitamin D binding protein (DBP) and vitamin D receptor (VDR), sunlight exposure, and the effect of vitamin D supplementation on weight gain also need to be considered.

## Competing interests

The authors had no conflict of interest.

## Authors' contributions

MD contributed to the study design and supervised in biochemistry experiments; AS, ET and AMM carried out the experiments and provided the manuscript; MQ involved in the data analysis and the interpretation of results. All authors have read and approved the content of the manuscript.
